# The Role of Self-regulation and Affective Control in Predicting Interpersonal Reactivity of Drug Addicts

**DOI:** 10.5812/ijhrba.9919

**Published:** 2013-06-26

**Authors:** Abbas Abolghasemi, Saeed Rajabi

**Affiliations:** 1 Department of Psychology, Faculty of Education and Psychology, University of Mohaghegh Ardabili, Ardebil, IR Iran

**Keywords:** Behavior, Addictive, Affective, Drug Addicts

## Abstract

**Background:**

Due to its progressive nature in all aspects of life, addiction endangers the health of individuals, families and the society.

**Objectives:**

The purpose of this study was to determine the role of self-regulation and affective control in predicting interpersonal reactivity of drug addicts.

**Materials and Methods:**

This research is a correlation study. The statistical population of this study includes all drug addicts who were referred to addiction treatment centers of Ardabil in 2011 of whom 160 addicts were selected through convenience sampling. A self-regulation questionnaire, interpersonal reactivity questionnaire and affective control scale were used for data collection.

**Results:**

Research results showed that self-regulation (r = -0.40) and affective control (r = -0.29) have a significant relationship with interpersonal reactivity of addicts (P < 0.001). The results of the multiple regression analysis indicated that 19 percent of interpersonal reactivity can be predicted by self-regulation and affective control.

**Conclusion:**

These results suggest that self-regulation and affective control play an important role in exacerbating as well as reducing interpersonal reactivity of addicts.

## 1. Background

Due to its progressive nature in all aspects of life, addiction endangers the health of individuals, families and the society. Substance abuse (e.g. stimulants and psychotropic substances) has increased in recent years in Iran due to a lack of cultural and social considerations as well as inefficiency of prevention programs (1). A prevalence of 800,000 to 2.5 million addicts, four times increase in the number of addicts of drug rehabilitation centers during 1983 to 1996 and 3 to 6 % incidence of addiction in Iran verify this claim. On the other hand, 90% relapse of addiction after quitting in Iran indicates that many more attempts must be made for understanding the causes of propensity to this predicament and identifying effective ways to deal with it (2). One of the important factors affecting substance abuse is interpersonal reactivity. People who cannot control their excitements are possibly more at risk of substance abuse (3). Excitement and impulsiveness are personality characteristics of those who demonstrate high reactivity toward life events. These are among the factors that affect problematic behaviors, especially substance abuse. Chassin et al. and Blackson et al. found that emotional reactivity is associated with early drug abuse. They concluded that emotional reactivity is a risk factor for starting substance abuse in the future (4, 5). In a research, Fox et al. found that drug abusers have many difficulties in regulating, understanding and managing emotions and in controlling impulsivity, especially at early stages of tendency to drug (6). One of the factors affecting the interpersonal reactivity of addicts is self- regulation. Some researchers argue that self- regulation (7) and interpersonal reactivity (3) can be influenced by drugs. Self-regulation requires controlling of conscious efforts and the ability to act on self-guided arrangements away from any internal reward and support. This is essential for recognition, monitoring and changes of behaviors versus environmental changes (8). Self-regulation is considered as mental efforts in monitoring internal conditions, processes and functions in order to achieve higher goals (9). In a research, Glassman et al. showed that self-regulation strategies are the best predictor of alcohol consumption, such that self-regulation refrains people from alcohol consumption (10). In their research, Doran et al. found that those who have low self-regulation have less ability to predict others’ needs and are often drawn towards smoking (3). In a research on 787 drug addicts, Cole et al. concluded that emotion, cognition and behavior derived from low self-regulation, could predict substance abuse disorders (9). Due to their family history, these people are more at risk of substance abuse. Therefore, failure in self-regulation plays an important role in relapse of drug abuse. In their research, Abolghasemi et al. concluded that drug abusers with high reactivity bring into play more negative emotion regulation strategies (11). Another variable that can influence the interpersonal reactivity of addicts is affective control. Studies have shown that inadequate emotional growth, difficulty in organizing behavior and having negative excitements are among the characteristics of drug-prone people (12). Copeland et al. showed that 71% of methamphetamine abusers received diagnosis of mood disorders, and 27% received diagnosis of anxiety disorders (13). Pitts et al. found that the effects of stimulants on self-control behaviors may be due to a reduced sensitivity of behavioral reinforcements (14). Vik showed that there is a positive relationship between methamphetamine abuse and psychiatric disorders (15). In a study, Parker et al. found that the failure to establish an emotional relationship with others results in drug abuse (16). Research evidence have indicated that use of methamphetamine is associated with depression (17), antisocial behaviors and depression (18), psychosis damages and anxiety (19) and low self-control and depression (20). The research results of Cole et al. also showed that self-control and personal control have a negative relationship with received stress in drug abuse (9). Given that the role of these variables are not appropriately highlighted and due to excessive tendency of the younger generation towards stimulant drugs as well as the importance of self-regulation and affective control in this age group and considering research gaps in this area, conducting this study is important and necessary. The aim of this study was to determine the role of self-regulation and affective control in predicting interpersonal reactivity of drug and psychotropic drug addicts.

## 2. Objectives

The purpose of this study was to determine the role of self-regulation and affective control in predicting interpersonal reactivity of drug addicts.

## 3. Materials and Methods

This research was an analytical cross-sectional study and used a correlation study design. The statistical population of this study included all drug addicts who were referred to addiction treatment centers of Ardabil in 2011, of whom 160 addicts were selected through available sampling. Inclusion criteria for the selection of subjects were: addiction to drug abuse, being male, married, and age range from 20 to 39 years and having an education higher than elementary school. Exclusion criteria were: suffering from one of the psychotic disorders, bipolar or dissociative disorder, and suffering from any severe medical disease. The following tools were used for data collection:

### 3.1. Self-regulation Questionnaire

The self-regulation questionnaire was designed by Brown et al. (1999) to assess the self-regulation processes. It has 63 items made to measure the overall ability to regulate behavior. Each item can be responded based on a 5-degree Likert Scale (strongly disagree, disagree, not sure, agree, strongly agree). Internal consistency and test-retest reliability coefficients of this questionnaire were 0.91 and 0.94, respectively. In the study of Aubrey et al. (1994), this scale had a negative correlation with the intensity of alcohol consumption (r = -0.23) and the consequences of drinking alcohol (r = -0.23) (P < 0.01) (21). Cronbach’s alpha coefficient of this questionnaire was reported to be 0.82 in Tayebi’s research (22).

### 3.2. Affective Control Scale 

This scale was designed by Williams et al. (23) and contains 42 items and 4 subscales (anger, depressed mood, anxiety and positive emotion). Each item can be responded based on a 7-degree Likert Scale (totally disagree to totally agree). Cronbach’s alpha coefficient of this test in the Williams et al. study was 0.94 and 0.72 to 0.91 for its subscales. Also, test-retest reliability coefficient of this scale after 2 weeks was reported as 0.78 for the entire scale and 0.66 to 0.77 for the subscales. Discriminant validity coefficient of this scale was obtained using Marlow-Crowne social desirability index (r = -0.17). Convergent validity of this scale in comparison with the Emotional Control Questionnaire was obtained to be -0.72 (P < 0.001). Besides, the reliability coefficient of affective control scale was estimated as 0.84 by Ghaderi et al. (24).

### 3.3. Interpersonal Reactivity Index

Interpersonal reactivity index was designed to measure interpersonal behaviors (25). This questionnaire has 28 items, each of which can be responded based on a five-option scale (“it does not describe me very well” to “it describes me very well”). Cronbach’s alpha coefficient for this index ranges from 0.75 to 0.82 (25). Cronbach’s alpha coefficient and test-retest reliability coefficient of drug addicts were 0.77 and 0.76, respectively. In addition, Allah Qalilou (26), found that there is a significant difference between drug abusers and normal individuals in terms of interpersonal behavior scale (P < 0.01).

First, necessary arrangements were carried out with the relevant authorities in the health and drug treatment centers of Ardebil to perform the research and collect the data. Then, the patients were referred to a psychologist room (research performer). After earning their trust and encouraging them to cooperate through the interview, they were asked to accurately answer the questions. They were assured that the results would be kept confidential and their information would only be used in the research. After observing the inclusion and exclusion criteria and giving the necessary instructions, questionnaires were given to them. Finally, the research data were analyzed by statistical methods including Pearson correlation coefficient and multivariate regression analysis using the SPSS software.

## 4. Results

The age mean and standard deviation of addicts were 39.8 and 4.45 respectively with a range of 29 - 40 years. 48.8% of drug addicts had elementary school education, 30.6% diploma and 20.6% associate degree or higher. 11.9% of addicts were employees, 51.88% were self-employed and 36.3% were unemployed. 23.1% of addicts reported that one or more members of their family experienced substance abuse. Also, 57.7% of addicts reported a history of successive drug quitting.

**Table 1:** Means, Standard Deviations and Coefficients of Correlation of Self-regulation, Affective Control and Interpersonal Reactivity of Drug Addicts

Variable	Mean ± SD	Interpersonal Reactivity
**Self- regulation**		
Receiving relevant information	25.67 ± 5.75	-0.30^c^
Evaluating the information and comparing it to norms	27.61 ± 4.13	-0.12
Triggering change	29.82 ± 3.52	-0.27^b^
Searching for options	30.39 ± 4.21	0.011
Formulating a plan	27.02 ± 5.43	-0.50^c^
Implementing the plan	28.69 ± 4.78	-0.37^c^
Assessing the plan's effectiveness	28.51 ± 3.73	-0.18^a^
**Total**	2.02 ± 20.78	-0.40^c^
**Affective control**		
Anger	31.40 ± 6.53	-0.25^b^
Positive affect	54.73 ± 9.10	-0.37^c^
Depressed mood	31.29 ± 6.26	-0.39^c^
Anxiety	52.49 ± 12.24	-0.36^c^
**Total**	36.26 ± 9.10	0.37^c^
**Interpersonal reactivity**	16.40 ± 3.86	1

^a^P < 0.05

^b^P < 0.01

^c^P < 0.001

As seen in Table 1, in drug addicts the (Mean ± SD) of self-regulation score affective control and interpersonal reactivity (2.02 ± 20.78), (36.26 ± 9.10), and (16.40 ± 3.86) respectively. The results of Pearson correlation indicated that self-regulation (r = -0.40) and affective control (r = 0.37) had respectively significant negative and positive relationship with the interpersonal reactivity of addicts (P < 0.001).

**Table 2.** Summary of Multivariate Regression Analysis Results for Prediction of Interpersonal Reactivity of Addicts Through Self-regulation and Affective Control Variables[Table-fn fn5313]

Predictor Variables	MR	RS	F	Nonstandard Coefficients		β	T
				b	SE		
**Constant**	-	-	-	23.47	3.61	-	6.50^b^
**Self-regulation**	0.397	0.158	29.54^b^	-0.074	.014	-0.397	-5.43^b^
**Affective control **	0.458	0.210	20.83^b^	0.107	.033	0.252	3.22^a^

Abbreviations: MR; multiple relation, RS; relation square, F; f-ratio, SE; Standard Error β; Beta, T; t-ratio

^a^P < 0.01

^b^P < 0.001

To determine the effect of each variable, self-regulation and affective control as predictor variables and interpersonal reactivity of addicts as a criterion variable were analyzed using multiple regression analysis through the entry method. As seen in Table 2, the results indicate that 21% of the interpersonal reactivity variance is defined by self-regulation and affective control variables. According to the beta values, self-regulation (β = -.397) and affective control (β = 0.252) are respectively the strongest variables to predict reactivity in addicts.

**Table 3.** Summary of Multivariate Stepwise Regression Analysis Results for Prediction of Interpersonal Reactivity of Addicts Through Self-regulation and Affective Control Variables[Table-fn fn5911][Table-fn fn5314]

Predictor Variables	b	SE	β	T
**Constant**	32.32	2.75	-	11.67^b^
**Formulating a plan**	-0.356	0.049	-0.500	-7.27^b^
**Assessing the plan's effectiveness**	-0.188	0.070	-0.182	-2.69^a^
**Depressed mood**	-0.153	0.047	-0.248	-3.27^a^
**Searching for options**	0.166	0.063	0.182	2.63^a^

Abbreviations: SE; Standard Error β; Beta, T; t-ratio

Explanation: First step: (R2 = 0.250; F = 52.80); Second step: (R2 = 0.283; F = 31.05); Third step: (R2 = 0.329; F = 25.55); Fourth step: (R2 = 0.358; F = 21.62).

^a^P < 0.01

^b^P < 0.001

Among the aspects of self-regulation and affective control, plan formulation, measurement evaluation, depressed mood and search of conditions were entered into the equation during the four steps to explain interpersonal reactivity of addicts. The coefficients of plan formulation, measurement evaluation, depressed mood and search of conditions to explain interpersonal reactivity of addicts were significant (P < 0.01). In general, the regression equation for predicting interpersonal reactivity of addicts at the final step was as follows: (Plan Formulation) (-0.356) + (Measurement evaluation) (-0.188) + (Depressed mood) (-0.153) + (Search of conditions) (0.166) + 32.32 = Interpersonal reactivity of addicts

## 5. Discussion

The aim of this study was to determine the role of self-regulation and affective control in predicting interpersonal reactivity of addicts. The results indicated that there is a significant relationship between self-regulation and interpersonal reactivity of addicts. In other words, the addicts who have low self-regulation would experience high personal distress and have a negative outlook about the future. These findings are in line with the studies of Glassman et al. (10), Doran et al. (3) and Abolghasemi et al. (11). The people who have high self-regulation also have a better performance in their social life and consequently experience fewer complications and problems. In contrast, low self-regulation will result in inclination of people to substance abuse, which leads to problems in interpersonal behaviors and social relationships. It will also shape the belief that they are isolated and abandoned by the society and they will turn to deviant behaviors instead of performing internally planned behaviors. This will keep such people in a low self-regulation condition and disable them from controling their conduct according to social standards. Eventually, they would not have constructive relationships in the society. Another part of the results suggested that there is a significant relationship between affective control and interpersonal reactivity of addicts. This is in line with the studies of Dawes et al. (12), Parker et al. (16), Otten et al. (20) and Cole et al. (9). In explaining these findings, it can be stated that addicts probably engage in substance abuse to cope with unpleasant sensations or to reduce negative emotions. On the other hand, it seems that they do not have necessary social skills to avoid substance abuse. Since they have a limited ability to evaluate long-term interests (lack of cognitive foresight), they cannot perform reasonable behaviors and will face problems in social relationships and interpersonal behaviors. The results also indicated that self-regulation and affective control significantly explain interpersonal reactivity of addicts. The role of these variables in predicting interpersonal reactivity of addicts is about 21%, (P < 0.05) and the remaining factors of interpersonal reactivity of drug abusers is 79% which can be explained by other variables (e.g. other cognitive and emotional factors and biochemical parameters, etc.). In explaining these findings, it can be said that self-regulation is essential for identifying, reviewing and changing behavior (8). In fact, people with high self-regulation encounter fewer emotional problems such as depression. These people are able to control their impulsive behaviors and thus do more acceptable behaviors in the society, which results in high social interactions in their life. People with low self-regulation are unable to effectively address their emotions and to manage them and poor management of their emotions increases the risk of substance abuse (16). In fact, regulating and managing excitements will make the individual apply appropriate coping strategies in situations where a person is at high risk of substance abuse and hence resist further against drug abuse. Affective control can also cause a person to perform well at interpersonal behaviors and to have the ability to control impulses and emotions. Consequently, this affective control makes people have more rational behaviors, a better understanding of issues and higher social skills and proceed more successfully at interpersonal behaviors. The results of multiple regression analysis showed that the components of self-regulation and affective control significantly explain interpersonal reactivity of addicts. The role of these variables in predicting interpersonal reactivity of addicts was 36%. Among these components, plan formulation, measurement evaluation, depressed mood and search of conditions are considered the strongest variables for predicting interpersonal reactivity of addicts. That is the people with high self-regulation have higher mental ability to process social information. This capability can help people have a better understanding of the negative and adverse consequences of substance abuse and as a result act more successfully against social and psychological pressures for drug abuse (11). In fact, through proper formulation and processing of information and consideration of right and wrong behavior in different situations, these people can improve their social interactions. The addicts, however, do not have the required interpersonal and social skills due to lack of self-regulation and appropriate formulation of information. Consequently, they encounter many problems in their life, which gives them a sense of outrage with respect to the community that results in more isolation from the society and they will turn to deviant behaviors instead of doing internally planned behaviors. On the other hand, depressed mood in addicts is a tool to transform distressing emotions (such as anxiety, depression, anger and aggression). Substance abusers describe negative agitation emotions as unbearable and distressing and cannot manage emotional states without relying on drugs. Substance abusers use physiological and psychological properties of drugs to adjust and balance their negative emotions and to achieve emotional stability. In fact, such people relieve themselves by using preferred substance and their emotional states will become more tolerable for them (27). The limitation of this research was as follows: the sample was confined to male methadone addicts of Ardabil who were referred to treatment centers. This makes difficult the generalizability of results. It is recommended to conduct research on those who have not yet started methadone abuse. The results of this study also provide a theoretical framework for self-regulation and affective control training.
